# Maoism and mental illness: psychiatric institutionalization during the Chinese Cultural Revolution

**DOI:** 10.1177/0957154X221090631

**Published:** 2022-08-18

**Authors:** Emily Baum, Zhuyun Lin

**Affiliations:** University of California, Irvine, USA; The Hong Kong University of Science and Technology, Hong Kong

**Keywords:** China, Cultural Revolution, Mao Zedong, mental illness, psychiatry, schizophrenia

## Abstract

This article offers a preliminary analysis of psychiatric treatment during the Chinese Cultural Revolution on the basis of interviews and rare case records obtained from ‘F Hospital’ in southern China. In contrast to the prevailing view of psychiatry during this time, which highlights either rampant patient abuse or revolutionary ideology, we show that psychiatric treatment at this facility was not radically altered by the politics of the Maoist period. Instead, treatments were informed by a predominantly biomedical understanding of mental illness, one that derived from the prior training of the facility’s lead physicians. Although political education was nominally incorporated into patient rehabilitation and outpatient care, it was not a constitutive element of inpatient treatment during the acute phase of illness.

## Introduction

In recent years, historians have begun to turn their attention to the long-neglected subject of mental illness in China. Thanks to newly accessible archives and increasing interest in the history of public health, scholars have explored such topics as the rise of Chinese asylums and psychopathic hospitals, local idioms of distress, and the role played by foreign missionaries in instituting new vocabularies, treatments and psychiatric epistemologies (Baum, 2018; Li and Schmiedebach, 2015; Ma, 2014; Shapiro, 2014; Szto, 2014; Wang, 2019a; Wu and Wang, 2016). The vast majority of these studies have centred on the late Qing (1644–1911) and Republican (1911–49) periods, a time when Chinese intellectuals and statesmen were experimenting with the adoption and implementation of foreign modes of health delivery.

In contrast to the rich studies that have proliferated on the first half of the twentieth century, the period between 1949 and 1976 – an era marked by the establishment of the People’s Republic of China (PRC), the rise to power of the Chinese Communist Party (CCP), and the leadership of Mao Zedong – has been a rather barren terrain for historians of psychiatry. With only a few exceptions (Gao, 2015, 2019, 2020; Wang, 2019b), scholars have not made many inroads into exploring the vicissitudes of Chinese mental health practice during this time. This has been doubly true for the decade-long movement known as the Great Proletarian Cultural Revolution (1966–76), a period marked by brutal violence, the humiliation of intellectuals and political elites, and a descent in many regions onto the brink of anarchy. As a consequence of heightened censorship and the closure of related archives, our knowledge of psychiatric treatment during the Cultural Revolution has been, and continues to be, almost a complete blank (Huang and Kirsner, 2020: 6; Kleinman, 1988: 100; Pearson, 1991: 41).

To date, the Cultural Revolution has posed steep – and in some cases insurmountable – obstacles for those hoping to examine the contours of psychiatric study and treatment during the last years of the Maoist regime. In many extant studies, researchers either skip over this decade-long interval or patch together partial narratives on the basis of piecemeal and unreliable sources. Indeed, most of what we know about Cultural Revolution-era psychiatry has been comprised of two different but equally problematic streams of data: observations recorded by foreign ‘friends’ of the CCP who were invited on guided tours to China in the early 1970s (Kagan, 1972; Livingston and Lowinger, 1983: 86–98; Sidel, 1972, 1973; Sidel and Sidel, 1973: ch. 7), and later memoirs and testimonies, many of which were written by individuals who had suffered tremendously at the hands of the socialist state (Munro, 2002: 66–84, 2006: ch. 4; Wang, 2019b: 450–1).

Both of the above perspectives suffer from obvious biases, albeit of different kinds. As Veronica Pearson has pointed out, reports written by sympathetic foreigners ‘generally purveyed the party line, and were uncritical’. Moreover, because foreign medical delegations only toured two of the foremost psychiatric facilities in China – the Shanghai Number One Hospital and the Beijing Medical College Third Hospital – their observations ‘cannot be considered to be typical’ (Pearson, 2014: 158). Such was the case for the physician Victor Sidel and the psychiatric social worker Ruth Sidel, who had the opportunity to visit China as part of an officially sponsored delegation in 1971 and 1972. They remained generally optimistic at the state of mental healthcare under Mao Zedong, which they described as consisting of productive labour, reading Maoist teachings, generating a community ethos, and nurturing a sense of ‘revolutionary optimism’ (Sidel and Sidel, 1973: 162–3). Their observations would go on to shape the views of many like-minded contemporaries, who similarly championed the ‘new psychiatry’ emerging from communist China (e.g. Ho, 1974: 621).

Once the Cultural Revolution drew to a close, however, a flood of memoirs and ‘scar literature’ began to expose the abuses suffered by those who had been institutionalized under the Maoist regime. A 2002 report by Robin Munro, writing under the auspices of Human Rights Watch, provides one of the most detailed depictions of these abuses to date. As Munro argues, Cultural Revolution-era psychiatry quickly became the handmaiden of political persecution. Dissident or politically non-conformist individuals were frequently diagnosed as severely mentally ill and detained indefinitely in psychiatric wards. Meanwhile, patients who may have genuinely suffered from psychiatric disorders were released from the hospital once they voiced orthodox political views. In such an environment, ‘psychiatry and psychiatrists became superfluous’, and treatment was denied to those who needed it most. Those who refused to denounce their heterodox viewpoints, however, were forced to undergo struggle sessions, beatings and self-criticisms (Munro, 2002: 70–1; see also Munro, 2006).

There are certainly truths to both of these accounts, and the type of intervention a person received likely depended, at least to a certain extent, on the particulars of the individual case. Nevertheless, the fact that the above perspectives swing so far to the extremes of the ideological spectrum signals that there is a vast chasm that remains in the middle. Indeed, as the psychiatrists Sing Lee and Arthur Kleinman (2002: 121) have pointed out in response to Munro’s reports, the sheer size of the PRC and the breadth of its regional disparities means that psychiatric services ‘exhibit great variations in standard and quality’. It is therefore difficult, if not impossible, to characterize all of Maoist-era psychiatry with broad brushstrokes, be they positive or negative.

This article seeks a third route: one that explores the everyday realities of psychiatric treatment at a single hospital in southern China – one that we will refer to as ‘F Hospital’ – between the years 1969 and 1976. In contrast to the ideologically charged positions above, we show that psychiatrists and nurses at this facility pursued a far more mundane approach to mental healthcare than what has typically been ascribed to the Cultural Revolution period. Despite pressure to incorporate politics into their treatments, physicians at F Hospital only perfunctorily experimented with revolutionary approaches when they were compelled to do so by local officials. Instead, the standard regimen at the facility involved the administration of antipsychotic drugs, sometimes supplemented by Chinese herbal therapies and acupuncture. The biomedical orientation maintained by physicians at F Hospital meant that they tended to pursue medicinal treatments regardless of the form and perceived source of the mental disorder. By categorizing most types of mental illness as ‘schizophrenia’, the physicians legitimized the reality of the patient’s distress but downplayed potential psychosocial reasons for its onset.

Relying on evidence supplied by patient records and interviews with physicians and nurses, we argue that psychiatric treatment at F Hospital was neither radically altered nor driven to a halt by the Cultural Revolution. Although physicians remained under pressure to implement revolutionary class struggle, they continued to approach mental illness as a primarily ‘scientific’ problem, rather than an ideological one. Having been trained in psychiatric methods that viewed mental illness as a brain illness, their treatment modalities were a logical extension of the training they had received prior to the outbreak of the Cultural Revolution. Thus, while political education was nominally incorporated into patient rehabilitation and outpatient care, it was not a constitutive element of inpatient treatment during the acute phase of illness.

## F Hospital

F Hospital is located in a region that we will refer to as ‘X City’, a prefectural-level city in southern China.^1^ By the 1960s, X City had become a sizable market town and boasted a population of close to 300,000 people, making it one of the largest cities in the province. Its proximity to the biannual Guangzhou Export Commodities Fair (*Guang jiao hui*) also meant that it maintained a closer tie to foreign capital than most other regions in the country. Given the city’s significant distance from the capital of Beijing, those living in the area experienced the reverberations of the Cultural Revolution after a delay. During the months when the Commodities Fair was being held, moreover, local leaders may have helped attenuate some of the chaos of the movement in order to avert foreign attention (Vogel, 1969: 324–5). Nevertheless, X City was not immune to or isolated from the political ethos of the period, and physicians in the area remained under significant pressure to adhere to the revolutionary dictates of Mao Zedong Thought throughout the entirety of the Cultural Revolution.

Although F Hospital was not officially opened to the public until 1969, the original inspiration for its establishment can be traced to 1958, when the First National Conference on the Prevention of Mental Disorders (*Quanguo jingshen bing fangzhi gongzuo huiyi*) was held in Nanjing. At the conference, participants affirmed the need for more psychiatric institutions to be established in China (Cao, 1958), an appeal that was amplified when foundational psychiatrists such as Wu Zhengyi widely praised the accomplishments that had been achieved in the psychiatric field since the founding of the PRC in 1949 (Wu, 1959). Over the following years, medical workers in X City responded to the call for increased mental illness prevention work by proposing to build their own psychiatric institutions. Prior to this time, there were no such facilities in the area, and those with severe mental illnesses were sent to a well-known psychiatric hospital in a neighbouring city. Many others with psychiatric infirmities did not receive any institutional treatment at all, and instead could be found lingering on the street or under custodial care at home (Wu SL, 1989: 46).

In 1959, the local government of X City decided to start the construction of a sanatorium for the mentally ill (*jingshen bing liaoyang yuan*), which was mainly intended to accommodate homeless patients. The sanatorium was opened to the public in 1960 and remained in use until 1965, during which time it retained a modest population of around 60 patients, the majority of whom were itinerant and destitute (Wu SL, 1989: 46). In October 1966, the provincial government reassessed the situation and granted approval for a larger psychiatric hospital to be built. The construction lasted nearly one and a half years, and the main facilities – including one outpatient building, two medical wards, and living quarters for personnel and staff – were completed by the summer of 1968. Because of the upheaval caused by the onset of the Cultural Revolution, the opening of the hospital was delayed until the following year and it was not until 26 June 1969 that the hospital formally received its first patients (Jiang, 2008: 12).^2^

Despite these setbacks, preparations for the opening of the facility proceeded apace. All medical personnel were in residence and remunerated at a rate of 34 yuan per month even before the facility had begun admitting patients. Using a military metaphor, one medical worker observed that ‘food and fodder came ahead of the troops’ – that is, that preparations were made well in advance of the facility’s official opening (*bingma weidao, liangcao xianxing*) (Jiang, 2008: 14). According to a document issued by the municipal government in 1966, the original plan was for F Hospital to be equipped with 200 beds and a ratio of beds to staff of 1:0.6, though it did not quite reach this target by 1969.^3^ As an independent facility administered under the auspices of the local Bureau of Health, F Hospital was not affiliated with other academic institutions, and its lead physicians were therefore granted a certain degree of flexibility in determining the treatment modalities they employed.

One of the authors of the present study (Lin), a former social worker at F Hospital, was able to interview 10 medical workers from the first cohort of physicians and nurses, along with three other workers who joined the staff a few years later (see Table 1). On the basis of these interviews, it is possible to reconstruct a general picture of the background and experiences of F Hospital’s earliest employees. The first cohort assigned to F Hospital mainly comprised three categories: graduates from local medical schools, demobilized military personnel, and medical workers from other units. In the summer of 1967, 20 nurses and two physicians from local medical schools were the first to arrive on site. Several of these nurses, who had recently graduated from the medical school in X City, were part of the last cohort to finish their coursework before the outbreak of the Cultural Revolution (Nurse C, 2019).^4^ Yet despite their fairly comprehensive training, very few psychiatry courses were being offered in medical schools at the time, and nurses naturally expressed anxiety when they were notified that they would be dispatched to work at a psychiatric facility (Nurse L, 2018).

**Table 1.** Interviewees.

No.	Interviewee (pseudonym)	Age at interview*	Background before working	Educational level**	Position/Year of arrival
1	Nurse C	74	Local medical school	TSS diploma	Nurse/1967
2	Nurse L	73	Local medical school	TSS diploma	Nurse/1967
3	Nurse Y	75	Local medical school	TSS diploma	Nurse/1967
4	Nurse J	73	Local medical school	TSS diploma	Nurse/1967
5	Doctor G	76	Local medical school	TSS diploma	Doctor/1967
6	Doctor H	86	Psychiatric Prevention & Research Center of Guangdong	TSS diploma	Doctor/1967
7	Doctor Y	65	Demobilized soldier	Junior middle school	Doctor/1967
8	Pharmacist A	86	Local hospital	TSS diploma	Pharmacist/1970
9	Pharmacist B	62	Second cohort nurse; later transferred to pharmacy	Primary school	Pharmacist/1971
10	Pharmacist C	73	Provincial medical school	Unclear	Pharmacist/1968
11	Logistics worker A	74	Soldier	Primary school	Logistics worker/1968
12	Logistics worker B	74	Soldier	Primary school	Logistics worker/1968
13	Official Z	88	Local Bureau of Health	TSS diploma	Local official staff

* Most interviews were done in 2018 and 2019, with supplementary and follow-up interviews in 2020; **TSS = Technical secondary school.

The two recently-graduated physicians confronted a similar dilemma. Given that they lacked the necessary preparation to treat the mentally infirm, the local Bureau of Health arranged for them to receive six months of training at an established psychiatric facility in Guangzhou. During this time, they received instruction in psychiatric diagnosis and pharmacological treatments (Nurse C, 2020). Ultimately, the new cohort of medical workers was joined by an additional group of physicians, nurses and attendants, bringing the total number of staff at the hospital to 109 by the end of 1969. Among these, two senior physicians, known to be regional ‘experts’ in psychiatry, were placed in charge of the hospital’s medical activities, and they organized training sessions to further prepare the recent graduates for their new responsibilities. These senior experts took the lead in determining the treatment regimen that would be employed at the facility during their tenure (Jiang, 2008: 20).

In a series of interviews, medical workers recalled that their normal schedule prior to the opening of the hospital consisted of manual labour during the day and intensive study at night; the latter involved a combination of Maoist theory and psychiatric texts. In 1970, they were able to supplement their coursework with planned visits to other psychiatric facilities, both local and in regions such as Tianjin, Shanghai, Nanjing, Suzhou and Sichuan (Jiang, 2008: 19). According to one of our interviewees who toured these facilities, each hospital employed a somewhat different treatment regimen depending on the outlook of the physicians in charge. While some may have been able to covertly circumvent outside political pressures (Doctor H, 2021), others conformed to the officially sanctioned approach to Cultural Revolution-era psychiatry: namely, a combination of traditional Chinese medicine (TCM) and Mao Zedong Thought (Jia 2004; Sidel and Sidel, 1973). At one hospital in Hunan province, for example, one of our interviewees recalled that the primary treatment modality involved intensive political education and engagement in thought reform (Doctor H, 2019).

Despite great political pressure to adopt revolutionary methodologies, physicians at F Hospital decided to pursue a treatment regimen that consisted predominantly, if not wholly, of antipsychotic drugs. Their decision to do so was informed by a mixture of ideology and experience. As one of the senior psychiatric experts noted in an interview, the consensus at F Hospital was that mental illness was a scientific, rather than ideological, issue (Doctor H, 2019). Having received their medical training prior to the outbreak of the Cultural Revolution, they disagreed with therapeutic modalities that stressed the memorization and repetition of Mao Zedong Thought. At first, these physicians attempted to incorporate Chinese medicine more comprehensively into their treatment strategy, and they even invited famous TCM specialists to the hospital to attend to their patients. However, upon observing the treatment outcomes of herbal remedies, they concluded that TCM was not effective in mitigating the acute symptoms of mental illness, and they decided to incorporate herbs and acupuncture only as an adjunct to antipsychotic drugs (Doctor G, 2019; Nurse C, 2019; Nurse L, 2019).

We recognize that post-hoc testimonies about the Cultural Revolution period might be tainted due to lapses in memory, the experience of personal trauma, or the respondent’s decision to minimize or gloss over behaviours that would be considered deeply problematic by today’s standards. However, supporting evidence for the above claims can be found in the copious clinical records that remain at F Hospital today. After receiving approval from the Hospital’s Ethics Committee, one author (Lin) was given permission to access these case files, at first with the assistance of a research partner and later independently. In theory, the files were supposed to have been organized chronologically by the inpatient number recorded on the first page of the patient record. Yet in practice, the inpatient number did not always correspond to the date of admission, and many files were misplaced or out of order. For instance, the patients described in the first four numbered files were not admitted until the 1970s, while files 5 and 6 were admitted prior to the official opening of the hospital in June 1969. For these reasons, it was difficult to review the files in a systematic fashion and our original plan to read them in order was not feasible. In total, we reviewed 30 complete files comprising over 1100 pages of clinical materials, choosing them on the basis of the original inpatient number written on the first page of the case file.

Of the 30 patients discussed in the files, two-thirds were men. The mean age upon first admission was 30 years, with the youngest being 17 and the oldest 51. Occupations varied, but the majority were listed as farmers; other occupations included one student, one cadre (administrative official), and workers from other fields. More than half were unmarried. The class composition of the patient’s family was also recorded in some of the files, though there was no standard nomenclature for doing so and many had no information in this section. Fields were often left blank. For a complete list of demographic details included in the case files we reviewed, see Table 2.

**Table 2.** Data from 30 patient case files, F Hospital (1969–76).

Sex	Male 20	Female 10			
Marriage	Married 13	Unmarried 17			
Education	Lower primary school or 1-2 years elementary education 6	Higher primary school or 3–5 years elementary education 6	Junior middle school 2		
Occupation	Peasant 13	Worker* 10	Student 1	Other** 6	
Age	17–19: 3	20–29: 12	30–39: 10	40–49: 4	50+: 1
Class background	Poor peasant 11	Rich peasant 1	Worker 4	Peddler 2	Capitalist 1

* Unspecified; **Hairdresser, shopworker, office staff, cadre.

## Clinical approaches at F Hospital

By using case files to observe the ‘social and technical structure of contemporary healing’ (Risse and Warner, 1992: 185), it is possible to make some preliminary observations about what the normal treatment regimen, diagnostic process and overall approach to psychiatric institutionalization was like at this facility. Case files from F Hospital are lengthy and detailed, in some cases running upwards of 30 pages per admission. (Many patients were admitted to the hospital more than once.) They describe the process by which patients were received into the ward, examined by clinicians, diagnosed and evaluated, and treated before being discharged. On the whole, the records reveal that clinical approaches at the facility emphasized somatic aetiologies of mental illness and were not significantly influenced by the political situation of the Cultural Revolution.

When brought to the hospital, patients were first given two examinations: physical and mental. Physical examinations were rigorous and comprehensive, including a temperature check, analysis of the patient’s blood, liver, pulse, abdominal cavity, and breathing rate, and an otolaryngologic and ophthalmic examination. Physicians additionally tested the patients’ reflexes and questioned them about their nutritional intake. Perhaps because early PRC-era psychiatry tended to conceive of psychiatric problems as physical problems (Pearson, 1991: 32), physicians at F Hospital were highly attentive to bodily complaints. For instance, when one patient was brought to the hospital with a history of gastrointestinal problems, she was given Benactyzine^5^ to assuage it (Case 11); another patient was given a chest X-ray for suspected pneumonia (Case 30); and when a patient complained of experiencing a sore throat and nausea, she was given a laryngeal examination (Case 12).

After the physical examination was completed, patients underwent a psychiatric evaluation. Like the physical examination, the psychiatric evaluation was in some ways contingent on obtaining the patient’s cooperation. Physicians observed the patients’ behaviour and questioned them directly as a way to assess their ‘thinking’ (*siwei*), ‘consciousness’ (*zhijue*), and obstructions in their mental perception and awareness (*ganzhi*). The format of the examinations, at least as they were recorded in the case files, was formulaic. Physicians observed how the patients dressed (if their clothing was neat or unkempt), if they were able to walk into the ward unassisted and unchained, if they cooperated with the nurses and staff, and if their general demeanour was quiet or agitated, violent or subdued.

In addition to these generalized impressions, the most significant part of the psychiatric evaluation consisted of direct questioning. This was used as a way to ascertain the patient’s mental awareness and intellectual aptitude (*zhineng*). In most cases, the specific line of questioning was not included in the case files, though we can deduce that doctors typically asked the patients if they understood that they were sick and were currently being treated in a hospital. Occasionally, a more pointed line of questioning was included in the notes. In Case 12, for instance, a young woman was questioned as follows:

Doctor: How long were you in school?

Patient: Over two years.

Doctor: How long have you been working?

Patient: I’ve been working for over 100 years.

Despite the irregularity of the answer, the physician described the patient’s response as ‘keeping to the subject’ (*qieti*). He concluded that her short- and long-term memory still functioned but that it was hard to ascertain her level of ‘comprehension, calculation, and general knowledge’ because of difficulties in communication stemming from her unclear pronunciation.

Conversations and direct questioning were used as a way to gauge the quality of the patient’s speech (speed, content, fluency), their self-knowledge, ability to concentrate, and the content of their ideations. After admission to the hospital, doctor-patient conversations continued to take place within the ward. This type of assessment was considered to be a form of ‘passive interaction’ (*beidong jiechu*) since conversations were usually initiated by the doctors, and patients were often unwilling to speak about their illness or the content of their ‘hallucinations’ unless prodded. During such conversations, doctors were not just attentive to the content of the speech but also to the patient’s facial expressions, emotions, and behaviours. The most common description that appeared in the records was ‘affective flattening’ (*qinggan danmo*), which was interpreted as a significant marker of schizophrenia.

All of this information was taken into consideration when physicians made their diagnoses. With only two exceptions, each of the 30 patients under consideration were diagnosed with some variant of schizophrenia: either hebephrenia (incoherent and disorganized schizophrenia), paranoid schizophrenia, or dissociative schizophrenia. The remaining two patients were diagnosed with senile psychosis (Case 24) and mental illness resulting from alcohol or drug poisoning (Case 27). In some instances, physicians noted the uncertainty of their preliminary diagnosis by including a question mark in the case file; these diagnoses were typically confirmed upon further observation and were only challenged on very rare occasions.

A diagnosis of schizophrenia naturally informed the treatment regimen. Chlorpromazine, an antipsychotic, was the primary drug used in the hospital, sometimes employed alongside other antipsychotics like perphenazine, Taractan (a brand of chlorprothixene) and Largactil (a brand of chlorpromazine). Other drugs that were frequently mentioned included Phenergan (an antihistamine and sedative), Amytal (a barbiturate), Secobarbital (a sleep aid), Hyminal (a brand of methaqualone, a hypnotic and sedative) and hyoscine (an anti-nausea medication). Physicians sometimes noted these drugs in English or used their Chinese name or an acronym. In some instances, the patient’s underlying physical condition influenced the type of medication given. One patient, a 27-year-old woman, was found to have a high level of the transaminase enzyme in her liver, a condition that made it dangerous to administer chlorpromazine. To bypass the problem, her doctors decided to use a combination of perphenazine and Phenergan, which were less harmful to the liver but less effective in treating the symptoms of schizophrenia. In her case file, a physician admitted that ‘without chlorpromazine, her mental status will not improve, but using it will damage her liver. There is [therefore] a conflict in her treatment’ (Case 19).

By reading case files as a whole, it becomes possible to challenge some long-standing misconceptions about psychiatric treatment during the Cultural Revolution. Extant studies, citing either ideological reasons or limited access to Western drugs, suggest that psychiatrists prioritized Chinese medicine over biomedicine as a cure for mental illness during this period (Xia and Zhang, 1987: 27; Xu, 1995). Although Chinese medical treatments – including acupuncture, electroacupuncture, moxibustion, and herbal therapies – do appear in the case records, these types of treatments were mainly used as an adjunct to antipsychotic medications. Moreover, in interviews, physicians noted that acupuncture and herbal treatments had little effect on the acute symptoms of mental disorder; while patients on an antipsychotic regimen typically showed improvement within two weeks, those on Chinese medicine did not (Doctor H, 2019). In one case, physicians observed how Chinese therapies could be actively detrimental to the patient’s health and recovery. In 1973, a patient at F Hospital was treated by a famous TCM specialist who performed moxibustion on the patient’s glans penis. Not only did this have no effect on the patient’s mental health, but it caused the glans to fester (Case 29; Doctor H, 2019). In this case, as with several others, the physicians decided to halt the traditional regimen and revert to using antipsychotic drugs exclusively (Cases 5, 6, 22).

Another misconception is that electroconvulsive therapy (ECT) and insulin shock therapy were either banned during the Cultural Revolution (Ho, 1974: 627; Pan, 2013; Sidel and Sidel, 1973: 163; Xia and Zhang, 1987: 26) or used as punishment for political dissidents (Munro, 2002: 59–60). Records from F Hospital challenge both of these assessments; in case records, ECT and insulin therapy remained in use, but were only employed minimally and in cases when pharmaceutical treatments had failed. In Case 29, in which a 23-year-old man was admitted to the facility and diagnosed with paranoid schizophrenia, neither chlorpromazine nor moxibustion therapy showed any effect after nearly a month. Physicians decided to combine antipsychotics with ECT, which they administered over 10 sessions, and noted a positive outcome. Likewise, Case 15, who was admitted to the hospital nine times over a five-year period, was treated with insulin shock therapy on his sixth admission, but with no noticeable improvement.

From the above discussion, we can surmise that the treatment of patients at F Hospital was not determined strictly by political ideology but rather by clinical efficacy and the personal viewpoints of the facility’s lead physicians. Despite the fact that Mao Zedong Thought was widely propagated as a cure for mental illness during the Cultural Revolution (Hunan sheng, 1972), medical workers at F Hospital minimized the use of such approaches because they did not view them as effective. As Doctor H explained in an interview, he disagreed with the idea that mental illness should be considered a ‘thought problem’ (*sixiang wenti*) and persisted in approaching it as a ‘natural science’ (*ziran kexue*). He became more resolute in his stance upon visiting a hospital in Hunan province as part of his professional training:

I went to Hunan for advanced studies [in 1970]. They approached mental illness as a thought problem. I told you that I didn’t agree with this. [When] the patients had delusions of persecution, you [the medical workers] had to eat their shit to prove they weren’t poisoned. I couldn’t do it. I thought it was a scientific issue, not a thought issue. Hence we didn’t learn from them. We insisted on using Western medicine [antipsychotic drugs] to treat [the patients]. (Doctor H, 2019)

Other medical workers at F Hospital separately confirmed that they did not employ politically inflected therapies during the acute phase of illness (Nurse L, 2018, 2021; Nurse Y, 2021; Pharmacist B, 2021). Nurse L (2018) brushed aside questions about using ‘thought education’ on psychiatric patients by explaining that such a method would have been like ‘playing the lute to a cow’ (*duiniu tanqin*) – that is, directed towards the entirely wrong audience. The hospital’s case files, which include little evidence of revolutionary rhetoric, support such statements. Only on two occasions did references to ideology appear in the clinical records we reviewed: in Cases 7 and 8, two patients were advised to ‘pragmatically learn and apply Mao Zedong Thought’ (*huoxue huoyong Mao Zedong sixiang*) as part of their outpatient treatment plan.

## Politics and trauma

While revolutionary Maoism may not have been central to F Hospital’s therapeutic strategy, politics was never absent from the life of the facility. The upheaval taking place beyond the walls of the institution inevitably seeped inside, and medical workers were forced to respond to these political exigencies in ways that satisfied the demands of local authorities. In some ways, F Hospital’s geographical location in the south of China and its establishment in 1969 may have shielded it from the peak of revolutionary violence, which largely occurred between the years 1966 and 1968. Nevertheless, throughout the latter half of the Cultural Revolution period, physicians and nurses at the facility were still compelled by the nationwide imperative to participate in revolutionary class struggle. Thus, while the absence of Maoism in the written record bolsters the biomedical outlook emphasized in our interview data, it also suggests that the files themselves do not reflect a comprehensive picture of institutional life.

The periodic intervention of outside authorities was one aspect of life at F Hospital that only became apparent through unpublished memoirs and the recollections of our interviewees. Occasionally, the local Public Security Bureau visited the hospital and accused patients of being counter-revolutionaries or ‘rebels’ (*zaofan pai*), a charge that had serious implications even for those who were undergoing psychiatric treatment (Jiang, 2008: 24). At one point, patients had written ‘topple Lin Biao’ on the floor with pieces of brick, and they were subsequently accused of counter-revolutionary behaviour.^6^ To mitigate the repercussions of this heterodox action, medical workers were compelled to conduct a ‘struggle session’ in the ward – an activity that, at least in retrospect, they felt deeply conflicted about: ‘We [the physicians] knew that their misbehaviour was an expression of the mental illness. But if we didn’t follow the orders of the higher-ups, it would have been a mess (*zaogao*)’ (Doctor H, 2019).

Medical workers at F Hospital recognized that they could not maintain a pragmatically apolitical position without engendering risk for themselves or their patients. Indeed, the biomedical approach they had adopted conflicted with trends in contemporary Chinese psychiatry, which denied biological determinism in favour of a malleable and politically inflected understanding of the human psyche (Gao, 2020). To better conform to such a view without negating their own belief in the efficacy of scientific biomedicine, the leaders of F Hospital installed a ‘political thought guide’ (*zhengzhi sixiang zhidao yuan*) in both the male and female wards. The role of the thought guide was to engage patients in ‘heart-to-heart talks’ (*tanxin*) as part of the rehabilitative process. Such activities never appeared in the patient records we reviewed, however. The reason, according to our interviewees, was because political education was not considered a form of ‘treatment’ and was therefore applied only after the acute psychotic phase had already passed (Doctor H, 2021; Nurse L, 2021; Nurse Y, 2021; Pharmacist B, 2021).

While political ideology may have only been perfunctorily integrated into the treatment regimen in response to outside pressures, Maoist class struggle still lurked in the case files – but only insofar as it was considered a precipitating factor in the onset of psychotic symptoms. In many of the records we reviewed, families attributed the origin of the patient’s illness to the tumult, surveillance, and conformist pressures that accompanied the deepening of the Cultural Revolution. Yet, despite the perceived functional (that is, non-organic) source of the affliction, physicians at F Hospital typically diagnosed these patients as schizophrenic, thereby underscoring the somatic – rather than psychological or ideological – roots of the disorder. In so doing, medical workers legitimized the material reality of the patient’s condition but downplayed the psychosocial factors that may have contributed to its inception. The approach they adopted was therefore a double-edged sword: while it may have shielded their patients from the more destructive aspects of revolutionary class struggle, it also de-emphasized, if not neglected entirely, the traumas and psychological devastation that patients experienced as a result of Cultural Revolution politics (see Kleinman, 1986: 94).

As part of the standard admission procedure at F Hospital, patients (or often a family member or caretaker who spoke on their behalf) participated in an intake interview in which they were asked to describe the cause and course of the illness as they personally interpreted it. These descriptions were then recorded in the patient’s file under the headings ‘Precipitating Factors of Illness’ (*qibing youyin*) and ‘Current History’ (*xian bingshi*). According to these entries, the extreme political climate that characterized the Cultural Revolution could easily lead to psychological deterioration. For example, in June 1969, Mr T, a 35-year-old farmer, was chosen to be a member of his production team’s leadership group (*lingdao banzi*). As part of his new responsibilities, he was placed in charge of the team’s political study, which required him to read aloud from the *Selected Quotations of Chairman Mao*. Mr T agonized over his new duties, fearing that he would not be up to the task. As he told his wife, he was particularly nervous that his ‘low cultural level’ would cause him to make a mistake while reciting Mao’s words. He began to experience delusions, speak nonsensical words, develop paranoid thoughts that someone would hurt or murder him, and have suicidal ideations. He was eventually admitted to the hospital, where he was diagnosed with paranoid schizophrenia and treated with chlorpromazine and acupuncture to help him sleep. He was released from the facility exactly three months after admission (Case 13).

Another such example occurred in the case of Miss Z, a 23-year-old farmer. In 1969, she signed up to work at a farm called Red Flag. Her decision to do so, which she concealed from her parents, was probably inspired by the nationwide ‘Up to the Mountains, Down to the Countryside’ movement (*shangshan xiaxiang*), which was initiated in 1968 to bring urban youths to rural areas. Upon her arrival at Red Flag, she found that she was unsuited for the difficult work. Her distress was compounded when, six months later, she was informed that her father had died. She petitioned to be sent back to her original work unit, but her appeal was denied. In October 1970, she received word that her family’s house had collapsed and that her family wished for her to return home. For several days she tried to return her residence status (*hukou*) to her hometown but was not able to do so. Eventually, she developed insomnia and headaches, ‘laughed and cried for no reason’ and proclaimed that she had frequently slept with the village secretary and was pregnant with his child.^7^ She was admitted to F Hospital in February 1971 and was diagnosed with hebephrenic-type schizophrenia. She gradually improved when treated with chlorpromazine, Amitor (for headaches), and Meprobamate (for anxiety), and was released in May of the same year (Case 2).

A common theme in the case files was the fear of committing a political transgression and suffering its repercussions. In July 1969, a 23-year-old man named Mr Y was admitted to the hospital after refusing to perform for his production team and later misplacing a workbook of Chairman Mao’s quotations. His psychological state deteriorated when he was repeatedly forced to confess his ‘crimes’ to his production leader (Case 29). In Cases 4 and 5, likewise, both patients attributed the onset of their mental illness to having ‘talked back’ (*dingzui*) or ‘made a suggestion’ (*ti yijian*) to an authority figure, an action that led to the latter patient being ‘repressed’ (*shou yazhi*). For some time thereafter, both hallucinated that they were being targeted, that they or their families were going to be harmed or murdered, and that the leader was punishing them for their misbehaviours. This fear may have been grounded in personal experience. In Case 5, for instance, the patient’s illness history suggests that she may have first experienced psychiatric symptoms when her husband was ‘struggled against’ (that is, publicly denounced) at the outset of the Cultural Revolution. Later, during the ‘Clean Up the Ranks’ campaign in 1968, she relapsed when she witnessed people parading and holding placards on the street.

In at least one file we reviewed, a patient was accused of feigning his symptoms in order to achieve respite from the relentless pressures to participate in revolutionary Maoism. In July 1969, Mr L, a 41-year-old worker, was admitted for the first of what would eventually become six rounds of treatment. On the third day after his first admission, his work unit phoned the hospital with a pressing concern. Each time there was a national campaign or movement, they said, Mr L would experience psychotic symptoms. Once the campaign came to an end, however, his psychological state would return to normal. The work unit asked the physicians to surreptitiously determine whether or not Mr L was truly mentally ill. After convening a meeting to discuss the case, and after halting the further administration of Mr L’s antipsychotic drugs, physicians suspected that there may have been some manipulation but could not be completely certain (Case 25).

The above discussion is not meant to suggest that Cultural Revolution politics was the only, or even the most frequent, cause of mental distress among patients at F Hospital. Often, patients and their caretakers traced the onset of the illness to far more banal causes, including heartbreak and interpersonal crises (Cases 6, 7, 14, 16, 22, 23, 61) or the after-effects of a physical illness (Cases 3, 18, 20). In other instances, the illness seemed to arise spontaneously without any particular reason given (Cases 1, 24, 26). Nevertheless, the frequency with which the Cultural Revolution itself appeared as the ‘precipitating factor’ of the disorder is worthy of consideration. Similar to what the psychiatrist Arthur Kleinman (1986: 123–42) observed during consultations with Chinese patients in the 1980s, case records from F Hospital suggest that the political pressures and social upheaval occasioned by the Cultural Revolution were widely considered a source of mental distress. That the physicians at F Hospital neither challenged such an interpretation nor sought to remedy it through ideological means suggests that they were deliberately diverging from officially sanctioned approaches to psychiatric treatment, which centred on self-criticism, revolutionary optimism and a proper understanding of Mao Zedong Thought (Tianjin shi, 1975; Zhonghua shenjing jingshen ke, 1966). In this sense, there may have been less political coercion at institutions such as F Hospital than was previously suspected.

By diagnosing patients as schizophrenic – and treating them as such through purely medicinal interventions – physicians legitimized the reality of the patients’ distress and offered them a temporary reprieve from outside political pressures. At the same time, however, by responding to all forms of mental illness with antipsychotic drugs, medical workers at F Hospital neglected to confront the psychological traumas that may have contributed to the onset of the disorder or exacerbated its pre-existing symptoms. The reason for this approach probably derived from contemporary exigencies. If, as Veronica Pearson has written, mental illness during the Cultural Revolution was conceived as ‘problems in thought, and therapy as thought liberation’ (Pearson, 1991: 46), then the only acceptable form of social intervention was class struggle. The scientific approach adopted by F Hospital’s physicians bypassed the more radical and combative modalities sanctioned by local authorities, but left few opportunities for potentially remedial psychotherapeutic care.

## Conclusion

Using case files and interviews from F Hospital in southern China, this article has provided a preliminary corrective to long-standing interpretations of Cultural Revolution-era psychiatry. While previous studies have stuck closely to either side of the political spectrum – charging psychiatric institutions with either abuse and maltreatment or an idealized Maoist-inflected revolutionary therapy – our data have shown that such assessments cannot be universally applied. At F Hospital, physicians extended the biomedical approach to treatment that they had learned prior to the outbreak of the Cultural Revolution. They consequently developed a therapeutic regimen that relied predominantly on the administration of antipsychotic drugs.

Admittedly, there are limits to the amount we are able to extrapolate from this evidence. The physicians at F Hospital acknowledged that their particular approach to treatment may not have been universally shared, and the geographical location of the hospital may have shielded it from some of the more violent aspects of Cultural Revolution struggle. Moreover, because F Hospital was not opened until 1969, it is impossible to gauge the extent to which the facility would have been affected by the peak of the Cultural Revolution, which lasted roughly from 1966 to 1968. By the time the hospital began admitting patients, much of the radical fervour of the movement had started to die down, although there continued to be periodic nationwide campaigns and ongoing pressure to conform to Maoist revolutionary ideology.

Regardless of these shortcomings, case records at F Hospital constitute an exceptionally illuminating look into the everyday realities of psychiatric treatment in the years leading up to Mao’s death. On the whole, they reveal that there may not have been as much political coercion, revolutionary ideology or outright abuse within certain Chinese psychiatric facilities as prior studies have suggested. As several interviewees independently confirmed, F Hospital was basically ‘quiet’ (*anjing*) throughout the early years of its operation, and its quotidian approach to patient care remained predominantly biomedical in orientation (Logistics Worker B, 2019; Nurse C, 2019; Pharmacist B, 2018). Judging by the large number of cases that were admitted to the facility on more than one occasion, we can further speculate that families, caregivers, and work units deemed the institutional treatment offered at F Hospital to be at least temporarily useful, even if it was not permanently successful.

In contrast to the orthodox Party line that mental illness in revolutionary China was either the product of oppressive conditions in pre-1949 Chinese society or the result of politically incorrect thinking on the part of the patient (Kao, 1977: 60–1), case files from F Hospital demonstrate that – at least in the view of the patients and their caretakers – it was the Cultural Revolution itself that was a significant source of mental distress. While the facility’s biomedical approach may have helped to ease the outward symptoms of such psychological suffering, it could not address the more profound scars that had accrued as a result of the traumas its patients had experienced. These they would continue to bear long after the Cultural Revolution had drawn to a close.

## Notes

1. In order to maintain the anonymity of our sources and preserve the privacy of the individuals who appear in clinical records, we have chosen not to disclose the precise location of F Hospital or the names of physicians and staff.

2. *Wangshi jishi* [Record of past events]; unpublished personal recollections of a research subject; document held by the author.

3. X City Bureau of Health (1966) Guanyu zhuanqu jingshen bing yiyuan jijian jihua han [Regarding the construction plans of the prefectural psychiatric hospital]; document in F Hospital archives.

4. This and similar citations state the interviewee (see Table 1) and the year of the interview.

5. The Chinese name is *wei fukang*. While known in the USA as an antidepressant, Benactyzine was used for gastric problems in China and the Soviet Union.

6. In the early years of the Cultural Revolution, Lin Biao was named Mao’s chosen successor. He was later accused of plotting a coup against Mao in 1971.

7. One cannot help but wonder if there is some veracity to this statement, since the patient adamantly maintained it for several months before finally disavowing it. There is ample documentation of sent-down youths being raped or forced into unwanted relationships. See, for example: Honig, 2003; Honig and Zhao, 2019: 87–116; Yang, 1997: 4–5.
